# Simulated Gastrointestinal pH Condition Improves Antioxidant Properties of Wheat and Rice Flours

**DOI:** 10.3390/ijms13067496

**Published:** 2012-06-18

**Authors:** Kim Wei Chan, Nicholas M. H. Khong, Shahid Iqbal, Maznah Ismail

**Affiliations:** 1 Laboratory of Molecular Biomedicine, Institute of Bioscience, Universiti Putra Malaysia, 43400 UPM Serdang, Selangor Darul Ehsan, Malaysia; E-Mails: nmhkhong@gmail.com (N.M.H.K.); ranashahid313@gmail.com (S.I.); myhome.e@gmail.com (M.I.); 2 Department of Chemistry, University of Sargodha, Sargodha 40100, Pakistan

**Keywords:** wheat flour, rice flour, antioxidant activity, phenolic contents, simulated gastrointestinal pH

## Abstract

The present study was conducted to evaluate the antioxidant properties of wheat and rice flours under simulated gastrointestinal pH condition. After subjecting the wheat and rice flour slurries to simulated gastrointestinal pH condition, both slurries were centrifuged to obtain the crude phenolic extracts for further analyses. Extraction yield, total contents of phenolic and flavonoids were determined as such (untreated) and under simulated gastrointestinal pH condition (treated). 1,1-diphenyl-2-picrylhydrazyl radical (DPPH^•^) scavenging activity, 2,2′-azino-bis(3-ethylbenzthiazoline-6-sulphonic acid) radical cation (ABTS^•+^) scavenging activity, ferric reducing antioxidant power (FRAP), beta-carotene bleaching (BCB) and iron chelating activity assays were employed for the determination of antioxidant activity of the tested samples. In almost all of the assays performed, significant improvements in antioxidant properties (*p* < 0.05) were observed in both flours after treatment, suggesting that wheat and rice flours contain considerably heavy amounts of bound phenolics, and that their antioxidant properties might be improved under gastrointestinal digestive conditions.

## 1. Introduction

Epidemiological studies have revealed that diets rich in plant-based antioxidative compounds have a significant role in providing protection against a number of diseases associated with oxidative stress, which include cancers, cardiovascular and neurodegenerative diseases [1–3]. Coupled to disease preventive effects, their nutritional benefits and health promotional properties have prompted scientists, clinicians and researchers to explore and recommend foods with high antioxidant profiles. As a result of a series of investigations, a number of sources, based on their botanical origin, including fruits, vegetables, cereals, legumes, spices, herbs, and seeds, have been explored as potential sources of antioxidants [4–6]. But still the focus of research is to find the potential sources of antioxidants from daily routine dietary foods, as major part of world population cannot afford to take antioxidant supplements from additional dietary materials [7].

Wheat (*Triticum aestivum* L.) and rice (*Oryza sativa*) are two leading cereal crops of the world. Significant research has been done on the antioxidant and disease preventive potential of rice during the current quarter of this century and many promising results for the treatment of a number of ailments, including cancers and chronic disorders, have been obtained [8]. Rice is the second-largest cultivated crop globally, and almost 50% world’s population uses rice as staple food [9]. Though literature reports reveal that most of the antioxidative compounds like tocols and oryzanols are concentrated in the rice bran [10], no comprehensive reports discussing the antioxidant properties of rice flour/white rice could be found to the best of our knowledge. The antioxidant properties of white rice must be investigated, as white rice or rice flour are the main consumable products from rice crop. Literature reports reveal the presence of ferulic acid derivatives and some other bound phenolics in rice flour, which suggests that the investigation of its antioxidant attributes should be done under different pH conditions, as their extractability may be significantly influenced by the pH of the extraction medium [11].

Besides this, wheat is the most consumed cereal crop in the world. According to the International Grains Council, the total global production of wheat in 2009 was 681.9 million metric tons [12]. In many countries it is used as a staple routine food and its flour is used for making noodles and bakery products, such as breads and cakes. Literature reports reveal that wheat is among the richest sources of antioxidants, but, like rice, most of these are concentrated in the bran layer [13]. Most of the consumers use wheat flour after separating it from bran by sieving, and the bran is discarded. Wheat flour is reported to be a rich source of aleurones, ferulic acid, and phospholipids. It is rich in potentially bioactive compounds including phenolic acids, flavonoids, tocopherols, carotenoids and tannins besides many nutritional constituents including fiber, proteins, starch, *etc*. Many of the phenolics present in wheat flour, especially ferulic acid derivatives, are present in esterified forms [14]. Their bound structures restrict their digestion by endogenous enzymes and let them travel down to the colon in an undigested form, where they are reported to be absorbed and result in significant health promoting effects [15]. It is also reported that colon fermentation leads to conversion of bound phenolics to their free form, which are easily digestible here and are more beneficial for the body. Hence, their digestion is affected by the environment of the gastrointestinal tract [16]. Different studies have been reported on the release and digestion of a number of dietary materials in different parts of the gastrointestinal tract. For example, Dintzis and Watson, (1984) [17] reported that the binding of iron by wheat bran takes place to a significant extent at gastrointestinal pH. Similarly, Liyana-Pathirana and Shahidi (2005) [11], reported a significant increase in antioxidant activity of milling fractions from selected wheat varieties under gastrointestinal pH condition. Despite that, its consumption at such a major scale requires further investigation on its nutritional, phytochemical and health promising properties.

The main objective of this study was to evaluate the differences in antioxidant properties of wheat and rice flours in untreated *i.e.*, non-simulated, and treated *i.e.*, subjected to simulated gastrointestinal pH condition. As antioxidant activities are strongly dependent on the model system and assays employed, a wide range of assays following different principles were used. To the best of our knowledge, no comprehensive study on these materials has been presented so far. The study will provide a clearer picture of these materials for disease preventive and health promoting functions. The report might also serve as a milestone towards the formulation of functional foods and the development of nutraceuticals using wheat and rice flours.

## 2. Results and Discussion

### 2.1. Extraction Yield, TPC and TFC

Wheat (WF) and rice (RF) flours were extracted with deionized water at 37 °C, and yield of crude phenolic extracts were calculated as such (untreated) and under simulated gastrointestinal pH condition (treated). Results for extraction yield, total phenolic content (TPC) and total flavonoid content (TFC) of the tested samples are presented in Table 1. Simulated gastrointestinal pH adjustment significantly (*p* < 0.05) increased the extraction yield of WF and RF 1.93- and 2.61- fold, respectively. Such a massive increase in crude phenolic extraction yield suggests the possibility of the presence of bound phenolics in significant amounts in both WF and RF, which may get hydrolyzed into simpler or free forms under simulated gastrointestinal pH condition. These findings are in agreement with the observations of Zielinski and Kozlowska (2000) [18], who reported that the yield of extracts is strongly dependent on the method and conditions of extraction. A higher magnitude of change in yield of RF compared to WF is found after subjection to simulated gastrointestinal pH condition. This may be hypothesized to be due to the occurrence of higher amounts of bound/insoluble compounds in RF than WF, which could be released at lower pH condition.

Besides an increase in extraction yield, simulated gastrointestinal pH adjustment also concomitantly increased the phenolic content of both flours (*p* < 0.05). After subjection to simulated gastrointestinal pH adjustment, the TPC of WF increased marginally from 422.04 ± 3.24 to 424.67 ± 26.90 μg GAE/g defatted WF (*p* > 0.05), whereas, that of RF increased 1.58-fold *i.e.*, from 99.99 ± 0.71 to 157.91 ± 17.24 μg GAE/g defatted RF (*p* < 0.05). Both RF and WF contain derivatives of ferulic acid and some other phenolics, which are usually present in esterified forms and cannot be extracted and determined under normal condition. However, on subjection to low gastric pH, bound phenolics from both the flours were either released from their matrices or converted into the free form due to acid catalyzed hydrolysis. According to Pérez-Vicente *et al*. (2002) [19], phenolics are generally esterified in the form of sugars or acids and are released at gastrointestinal pH. Kim *et al*. (2006) [20], reported that the amount of bound phenolics in wheat was significantly higher than that of free phenolics, which could only be extracted by acid or base catalyzed hydrolysis. Furthermore, Adom *et al*. (2003) [21], also reported that the proportion of bound phenolics in wheat was 2.5- to 4.5-fold higher than that of free phenolics. Similar studies have been reported for some other cereals, but are lacking on RF and WF.

The total content of flavonoids for WF and RF increased 3.16- and 20.83-fold, respectively, after subjection to simulated gastrointestinal pH condition. The magnitude of increase in TFC, under gastrointestinal pH condition, was much higher than the TPC for both WF and RF. Flavonoids are polyphenolic compounds present in a number of dietary materials including cereals, and are reported to have appreciable antioxidant properties [22]. They have diverse structures depending on the hydroxyl groups around the aromatic ring, which also may affect their extractability in a particular medium. The rise in TFC, for both WF and RF, under simulated gastrointestinal pH condition, suggests that considerable amounts of bound flavonoids might have been present in WF and RF, which were later hydrolyzed and released from the matrices after subjection to low gastric pH. No study discussing the impact of gastrointestinal pH on changes in flavonoids content in WF and RF could be found.

### 2.2. Radical Scavenging Activity

**Figure 1:**
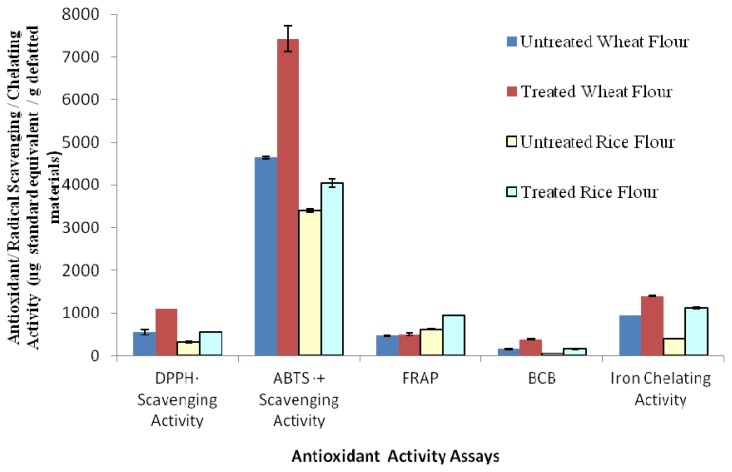
Antioxidant activities of wheat and rice flours as affected by simulated gastrointestinal pH condition. Results are obtained from means of three determinations ± standard deviation. Trolox was used as a standard in DPPH^•^ scavenging activity, ABTS^•+^ scavenging activity, ferric reducing antioxidant power (FRAP) and beta-carotene bleaching (BCB) assays. The standard for iron chelating activity assays was EDTA.

As number of diseases has been reported to be initiated or accelerated by different types of radicals, therefore scavenging of these radicals may result in control or prevention of these diseases. A large number of plant-based antioxidants have radical scavenging potential [22–24]. The radical scavenging potential of WF and RF was investigated using DPPH free radical (DPPH^•^) and ABTS radical cation (ABTS^•+^) as model substrates, and antioxidant activity was determined as the equivalent of Trolox *i.e.*, water-soluble analog of vitamin E. Results shown in Figure 1 demonstrate that both WF and RF exhibited appreciable DPPH^•^ and ABTS^•+^ scavenging activities, with difference being significant (*p* < 0.05) after subjection to simulated gastrointestinal pH condition. The increase in antioxidant activity of WF on the basis of DPPH radical and ABTS radical cation was 2- and 1.6-fold, respectively after pH adjustment, while it was 1.79- and 1.2-fold for RF (Figure 1). The antioxidant activity of WF, on the basis of DPPH radical scavenging, was significantly higher than that of RF before (1.75-fold) and after pH adjustment (1.95-fold). Similarly, antioxidant activity of WF measured on the basis of ABTS radical cation scavenging was 1.36-fold higher than RF before pH adjustment, while it was 1.83-fold higher after pH adjustment. The higher magnitude of antioxidant activity changes for WF and RF, based on both assays, after simulated gastrointestinal pH adjustment suggests that both the tested flours contained considerable amounts of bound antioxidative compounds (like phenolic compounds), which were then released under simulated gastrointestinal pH condition. Generally, the ABTS assay is preferred over the DPPH assay with the consideration that the ABTS assay gives consistent results over a wide pH range, while the viability of DPPH method is dependent on pH [25]. Moreover, ABTS can work equally well in organic or aqueous media and its efficiency is independent of the solvent system employed unlike the DPPH assay. In addition to this, the reaction of antioxidants with ABTS is quite fast and gets steady after almost 30 min, while the reaction with DPPH is much slower in comparison [26]. Overall findings are well in agreement with the results obtained for TPC and TFC assays.

### 2.3. Ferric Reducing Antioxidant Power (FRAP)

Ferric reducing antioxidant power (FRAP) is a well-established and recommended assay for the determination of antioxidant activity. The assay is simple, fast and reproducible [27]. Complex reactivity mechanisms of diverse reactive species present in plant extracts suggest determination of antioxidant activity through multiple assays, which may provide reproducible results. Well recognized reproducibility of the FRAP assay gives it superiority over others. FRAP for WF and RF was determined before and after simulation with gastrointestinal pH condition and the results were calculated as μg Trolox equivalent/g defatted material (Figure 1). The increment was 1.08- and 1.51-fold for WF and RF, respectively, after simulation. The FRAP was found to be 1.34-fold higher for RF than WF before simulation and 1.87-fold after simulation. The findings are in contradiction with other assays including TPC, TFC, DPPH and ABTS, which showed higher credentials for WF, as well as with earlier reports, which showed a direct relationship between FRAP and other assays [28]. This may be assumed to be due to the presence of certain compounds in RF, which might have a good reducing power for ferric ions, but are not active scavengers. Hence, these compounds additionally contributed in FRAP, and as a result its magnitude increased more than in WF.

### 2.4. Beta-Carotene Bleaching (BCB) Assay

The beta-carotene bleaching assay is among the more widely used ones for the determination of antioxidant activity, as β-carotene is the favorite substrate for free radical assisted oxidation [29]. Trolox was used as a standard and antioxidant activity for WF and RF was determined (Figure 1). After gastrointestinal pH adjustment, both WF and RF exhibited an appreciable increase in antioxidant activity measured on the basis of the extent of beta-carotene bleaching (*p* < 0.05) and the finding supported the results obtained on the basis of other assays. Wheat flour showed a significantly higher antioxidant activity than RF against linoleic acid oxidation, before and after the simulation treatments. In the case of WF, a 2.36-fold rise in antioxidant activity was observed after simulation under gastrointestinal pH condition, while the increase was 2.8-fold in case of RF. Before treatment, the antioxidant activity for WF was 2.83-fold higher than that for RF, while after treatment it was 2.37-fold higher. The data is in good correspondence with other antioxidant activity assays.

### 2.5. Iron Chelating Activity

Many transition metal ions act as prooxidants and catalyze oxidation of polyunsaturated fatty acids and other substrates. Ferrous ions are reported to be active in this regard; therefore ferrous ion chelating activity measurement is considered an important assay while determining the antioxidant properties of any extract [30]. The results for chelating activity were again in accordance with other assays; WF exhibited higher chelating activity than RF before and after simulation of gastrointestinal pH condition. The increase in chelating power of WF was 1.47-fold upon simulation, while it was 2.91-fold in case of RF (*p* < 0.05). Under non-simulated condition, chelating activity of WF was 2.47 times higher than for RF, while the after-simulation difference was one of 1.25-fold. The data again supports the findings of other assays, which demonstrated a higher magnitude of change for RF compared to WF after subjection to simulated gastrointestinal pH condition.

## 3. Experimental Section

### 3.1. Chemicals

The chemicals used in this study were of analytical or HPLC grade and includee: methanol, chloroform and Tween 20 (Fisher Scientific, Loughborough, Leicestershire, UK); linoleic acid, gallic acid, β-carotene (Type I synthetic, 95%), sodium bicarbonate, aluminium trichloride (AlCl_3_), potassium dihydrogen phosphate, dipotassium hydrogen phosphate, 6-hydroxy-2,5,7,8- tetramethylchroman-2-carboxylic acid (Trolox), 2,2′-azino-bis(3-ethylbenzthiazoline-6-sulphonic acid) (ABTS), potassium persulphate, ferrous chloride (FeCl_2_), ferric chloride (FeCl_3_), potassium ferricyanide [K_3_Fe(CN)_6_], ethylenediaminetetra acetic acid (EDTA), 1,1-diphenyl-2-picrylhydrazyl (DPPH), sodium hydroxide (NaOH), Folin-Ciocalteu’s phenol reagent and ferrozine (Sigma-Aldrich Co., St. Louis, MO, USA); *n*-hexane, trichloroacetic acid and hydrochloric acid (HCl) (Merck, Darmstadt, Germany).

### 3.2. Samples Preparation

Wheat (*Triticum aestivum* L.) and rice (*Oryza sativa*) flours, respectively were purchased from Srri Easwari Mills Sdn. Bhd, Shah Alam, Selangor, Malaysia and FFM Berhad, Klang, Selangor, Malaysia. Two hundred grams of each flour were homogenized (Ultra-turax T25 basic, IKA^®^-WERKE GmbH & Co. KG, Staufen, Germany) with 400 mL of *n*-hexane for 15 min at 9500 rpm. Subsequently, the mixture was filtered through Whatman No. 2 filter paper and the residues were re-extracted twice following the same procedure. Defatted flours were collected and dried in an oven at 50 °C for 3 h in order to remove the residual solvent. Finally, defatted flours were passed through a 30 mesh sieve and kept at −20 °C prior to the crude phenolics extraction procedure. The final fat content of the rice and wheat flours was 0.09 ± 0.02% and 0.14 ± 0.03%, respectively.

### 3.3. Crude Phenolics Extraction

Crude phenolics of defatted flours were extracted according to an already described procedure [14]. In brief, 10 g of defatted samples were continuously stirred for 30 min with 150 mL of deionised water. The slurries obtained were centrifuged at 4500 rpm for 30 min at ambient temperature. The resulting supernatants were filtered through Whatman No. 1 filter paper and lyophilized (Virtis Benchtop K Freeze Dryer, SP Industries, Warminster, PA, USA) to obtain the crude phenolic extract. Finally, the yield of crude extracts was measured before storing at −80 °C for further analyses.

In order to determine the effect of simulated gastrointestinal pH condition on phenolic content and antioxidant activity of the tested samples, 10 g of defatted flours were continuously stirred for 30 min with 150 mL of deionised water. Then, the mixtures were acidified to pH 2 using 6 N HCl and incubated at 37 °C for 30 min. Subsequently after incubation, the pH was raised to 7 using 4 N NaOH and samples were incubated for a further 30 min at 37 °C. Finally, the treated samples were centrifuged, filtered, lyophilized and stored under the same condition as the untreated samples.

### 3.4. Total Phenolic and Flavonoid Contents

Total phenolic content (TPC) and flavonoid content (TFC) of the tested samples was determined as described previously [4].

Total phenolic content (TPC) of the tested samples was determined by Folin-Ciocalteu Reagent assay. In brief, 10 mg of crude phenolic extracts were individually dissolved in 1 mL of distilled water. Then, 0.1 mL of these solutions was serially reacted with 0.5 mL of 10% (*v/v*) Folin-Ciocalteu Reagent and 0.4 mL of 7.5% (*w/v*) sodium bicarbonate solution. After incubation at 40 °C for 90 min, 200 μL of the reaction mixture were loaded into a 96-well plate. The absorbance of the reaction mixtures was read at 765 nm using a microplate reader (Opsys MR™ 96-well microplate reader, Dynex Technologies, VA, USA). Gallic acid was used as standard and TPC of tested samples was expressed in micrograms gallic acid equivalents (μg GAE)/g defatted material.

Total flavonoid content (TFC) of the tested samples was determined using the aluminium calorimetric method. Generally, half a milliliter of crude phenolic extract solution was reacted with 2% (*w/v*) AlCl_3_ for 10 min in a 96-well plate. After that, the absorbance of the mixture was measured at 405 nm using a microplate reader (Opsys MR™ 96-well microplate reader, Dynex Technologies, VA, USA). The standard used in this assay was rutin, and TFC of the tested samples was expressed in micrograms of rutin equivalent (μg RE)/g defatted material.

### 3.5. Antioxidant Activity Assays

#### 3.5.1. DPPH^•^ Scavenging Activity

DPPH radical scavenging activity of the tested samples was determined as described previously [4]. In brief, 50 μL of crude phenolic extract solutions, in the respective concentration, were reacted with 195 μL of DPPH methanolic solution (0.1 mM) in a 96-well plate. Then the mixtures were swirled gently for 1 min. In order to minimize the evaporation of methanol from the mixtures, the microplate was covered with a lid and sealed with parafilm. The resulting mixtures were allowed to stand in the dark for 1 h and their absorbance was measured using a microplate reader (Opsys MR™ 96-well microplate reader, Dynex Technologies, VA, USA) at 540 nm. Trolox was used as standard and DPPH radical scavenging activity was expressed as μg Trolox equivalent/g defatted material.

#### 3.5.2. ABTS^•+^ Scavenging Activity

ABTS radical cation scavenging activity of the tested samples was determined according to the procedure described by Re *et al*. (1999) [31] with slight modifications. ABTS radical cation (ABTS^•+^) was produced by reacting 50 mL of 7 mM ABTS stock solution with 50 mL of 2.45 mM potassium persulfate for 24 h in the dark at room temperature. Then, the ABTS^•+^ solution was diluted with ethanol to an absorbance of 0.70 ± 0.02 at 734 nm (Pharmaspec UV-1700, Shimadzu, Kyoto, Japan). Subsequently, 950 μL of the adjusted solution were reacted with 50 μL of crude phenolic extract solution. The mixtures were vortexed and allowed to react in the dark at room temperature for 10 min. Finally, absorbance of the reaction mixtures was read at 734 nm using a spectrophotometer (Pharmaspec UV-1700, Shimadzu, Kyoto, Japan). Trolox was used as standard and ABTS radical cation scavenging activity was expressed as μg Trolox equivalent/g defatted material.

#### 3.5.3. Ferric Reducing Antioxidant Power (FRAP)

Ferric reducing antioxidant power (FRAP) of the tested samples was measured following the method of Oyaizu (1986) [32]. Initially, 1 mL of crude phenolic extracat solution was mixed with 2.5 mL of 0.2 M potassium phosphate buffer (pH 6.6) and 2.5 mL of 1% (*w/v*) potassium ferricyanide, respectively. The mixtures were incubated at 50 °C for 20 min followed by the addition of 10% (*w/v*) trichloroacetic acid (2.5 mL) and centrifugation at 3000 rpm for 10 min at ambient condition. Finally, 2.5 mL of the supernatant was diluted with an equal amount of distilled water and was reacted with 0.5 mL of FeCl_3_ (0.1%). Finally, the absorbance of the reaction mixtures was read at 593 nm using a spectrophotometer (Pharmaspec UV-1700, Shimadzu, Kyoto, Japan). Trolox was used as standard and FRAP of tested samples was expressed as μg Trolox equivalent/g defatted material.

#### 3.5.4. Beta-Carotene Bleaching Assay (BCB)

Antioxidant activity of the tested samples was evaluated by BCB assay, as described previously [4]. In brief, 3 mL of β-carotene solution (1 mg β-carotene/10 mL chloroform) were added to 120 mg of linoleic acid and 1200 mg of Tween 20. The mixture was mixed thoroughly and dried under a stream of nitrogen. Immediately, 100 mL of distilled water were added to the dried mixture to form a β-carotene-linoleic acid emulsion. In order to determine the antioxidant activity of the tested samples, 1.5 mL of the emulsion was added to 20 μL of crude phenolic extract solution, followed by incubation in a water bath at 50 °C for 1 h. Finally, the absorbance of the reaction mixtures was read at 470 nm using a spectrophotometer (Pharmaspec UV-1700, Shimadzu, Kyoto, Japan). Trolox was used as standard and antioxidant activity was expressed as μg Trolox equivalent/g defatted material.

#### 3.5.5. Iron Chelating Activity

Iron chelating activity of the tested samples was determined following a method described previously by Wang *et al*. (2009) [33]. Initially, 1 mL of each crude phenolic extract solution was mixed with 50 μL of 2 mM FeCl_2_ followed by the addition of ferrozine (100 μL; 5 mM) in each mixture. The mixtures were thoroughly vortexed and allowed to stand at room temperature for 10 min. After incubation, the absorbance of the resulting mixtures was measured at 562 nm spectrophotometrically (Pharmaspec UV-1700, Shimadzu, Kyoto, Japan). EDTA was used as standard and iron-chelating activity was expressed as μg EDTA equivalent/g defatted material.

### 3.6. Statistical Analysis

Data was reported as mean ± standard deviation from triplicate determinations. Analysis of variance (ANOVA) accompanied with LSD and Tukey tests (SPSS for Windows, Version 15) were conducted to identify the significant difference between the samples (*p* < 0.05).

## 4. Conclusions

Wheat and rice flours are promising sources of dietary antioxidants. The present study shows that simulated gastrointestinal pH adjustment significantly improves the phenolic content and antioxidant activity of WF and RF. This suggests that physiological condition factors such as gastrointestinal pH should always be taken into consideration, amongst other factors, for the determination of antioxidant activity, in order to get a clearer picture on antioxidant attributes of the ingested cereal-based flours in *in vivo* digestive tracts.

## Figures and Tables

**Table 1:** Extraction yield, total phenolic content (TPC) and total flavonoid content (TFC) of wheat and rice flours as affected by simulated gastrointestinal pH condition.

Flours	Wheat Flour		Rice Flour	
	
Tested Parameters	Untreated	Treated	Untreated	Treated
Extract yield (% *w/w*)	6.82 ± 0.43 a	13.16 ± 0.99 b	2.03 ± 0.01 c	5.32 ± 0.35 d
Total phenolic content (μg GAE/g defatted material)	422.04 ± 3.24 a	424.67 ± 26.90 a	99.99 ± 0.71 b	157.91 ± 17.24 c
Total flavonoid content (μg RE/g defatted material)	149.57 ± 26.78 a	472.39 ± 71.73 b	17.29 ± 0.20 c	360.17 ± 4.96 d

a–d: Results are obtained from means of three determinations ± standard deviation. Different alphabets within the same row indicate significant difference (*p* < 0.05).

## References

[1] Kastorini C.M., Milionis H.J., Esposito K., Giugliano D., Goudevenos J.A., Panagiotakos D.B. (2011). The effect of Mediterranean diet on metabolic syndrome and its components: A meta-analysis of 50 studies and 534,906 individuals. J. Am. Coll. Cardiol.

[2] Tydeman E.A., Parker M.L., Faulks R.M., Cross K.L., Fillery-Travis A., Gidley M.J., Rich G.T., Waldron K.W. (2010). Effect of Carrot (*Daucus carota*) microstructure on carotene bioaccessibility in the upper gastrointestinal tract. 2. *In vivo* digestions. J. Agric. Food Chem.

[3] Ismail M., Al-Naqeep G., Chan K.W. (2010). Nigella sativa thymoquinone-rich fraction greatly improves plasma antioxidant capacity and expression of antioxidant genes in hypercholesterolemic rats. Free Radic. Biol. Med.

[4] Ismail H.I., Chan K.W., Mariod A.A., Ismail M. (2010). Phenolic content and antioxidant activity of cantaloupe (*Cucumis melo*) methanolic extracts. Food Chem.

[5] Chan K.W., Ismail M. (2009). Supercritical carbon dioxide fluid extraction of *Hibiscus cannabinus* L. seed oil: A potential solvent-free and high antioxidative edible oil. Food Chem.

[6] Chan K.W., Khong N.M.H., Iqbal S., Ch’ng S.E., Babji A.S. (2012). Preparation of Clove buds Deodorized Aqueous Extract (CDAE) and evaluation of its potential to improve oxidative stability of chicken meatballs in comparison to synthetic and natural food antioxidants. J. Food Quality.

[7] Zhu K.X., Huang S., Peng W., Qian H.F., Zhou H.M. (2010). Effect of ultrafine grinding on hydration and antioxidant properties of wheat bran dietary fiber. Food Res. Int.

[8] Adom K.K., Liu R.H. (2002). Antioxidant activity of grains. J. Agric. Food Chem.

[9] Food and Agriculture Organization of the United Nations (2004). Food Outlook, No. 4.

[10] Iqbal S., Bhanger M., Anwar F. (2005). Antioxidant properties and components of some commercially available varieties of rice bran in Pakistan. Food Chem.

[11] Liyana-Pathirana C.M., Shahidi F. (2005). Antioxidant activity of commercial soft and hard wheat (*Triticum aestivum* L.) as affected by gastric pH conditions. J. Agric. Food Chem.

[12] International Grains Council (IGC) (2011). Grain Market Report.

[13] Iqbal S., Bhanger M., Anwar F. (2007). Antioxidant properties and components of bran extracts from selected wheat varieties commercially available in Pakistan. LWT-Food Sci. Technol.

[14] Baublis A., Decker E., Clydesdale F. (2000). Antioxidant effect of aqueous extracts from wheat based ready-to-eat breakfast cereals. Food Chem.

[15] Pérez-Jiménez J., Saura-Calixto F. (2005). Literature data may underestimate the actual antioxidant capacity of cereals. J. Agric. Food Chem.

[16] Liyana-Pathirana C.M., Shahidi F. (2007). The antioxidant potential of milling fractions from breadwheat and durum. J. Cereal Sci.

[17] Dintzis F.R., Watson P.R. (1984). Iron binding of wheat bran at human gastric pH. J. Agric. Food Chem.

[18] Zielinski H., Kozlowska H. (2000). Antioxidant activity and total phenolics in selected cereal grains and their different morphological fractions. J. Agric. Food Chem.

[19] Pérez-Vicente A., Gil-Izquierdo A., García-Viguera C. (2002). *In vitro* gastrointestinal digestion study of pomegranate juice phenolic compounds, anthocyanins, and vitamin C. J. Agric. Food Chem.

[20] Kim K.H., Tsao R., Yang R., Cui S.W. (2006). Phenolic acid profiles and antioxidant activities of wheat bran extracts and the effect of hydrolysis conditions. Food Chem.

[21] Adom K.K., Sorrells M.E., Liu R.H. (2003). Phytochemical profiles and antioxidant activity of wheat varieties. J. Agric. Food Chem.

[22] Shahidi F., Janitha P., Wanasundara P. (1992). Phenolic antioxidants. Crit. Rev. Food Sci. Nutr.

[23] Iqbal S., Younas U., Sirajuddin, Chan K.W., Sarfraz R.A., Uddin M.K. (2012). Proximate Composition and Antioxidant Potential of Leaves from Three Varieties of Mulberry (*Morus* spp.): A Comparative Study. Int. J. Mol. Sci.

[24] Iqbal S., Younas U., Chan K.W., Zia-Ul-Haq M., Ismail M. (2012). Chemical composition of *Artemisia annua* L. leaves and antioxidant potential of extracts as a function of extraction solvents. Molecules.

[25] Lemaska K., Szymusiak H., Tyrakowska B., Zieliski R., Soffers A.E.M.F., Rietjens I.M.C.M. (2001). The influence of pH on antioxidant properties and the mechanism of antioxidant action of hydroxyflavones. Free Radic. Biol. Med.

[26] Awika J.M., Rooney L.W., Wu X., Ronald L., Cisneros-Zevallos L. (2003). Screening methods to measure antioxidant activity of sorghum (*Sorghum bicolor*) and sorghum products. J. Agric. Food Chem.

[27] Saura-Calixto F., Pérez-Jiménez J., Goñi I. (2009). Contribution of cereals to dietary fibre and antioxidant intakes: Toward more reliable methodology. J Cereal Sci.

[28] Li H.B., Wong C.C., Cheng K.W., Chen F. (2008). Antioxidant properties *in vitro* and total phenolic contents in methanol extracts from medicinal plants. LWT-Food Sci. Technol.

[29] Pastore D., Trono D., Padalino L., Simone S., Valenti D., di Fonzo N., Passarella S. (2000). Inhibition by α-tocopherol and L-ascorbate of linoleate hydroperoxidation and β-carotene bleaching activities in durum wheat semolina. J. Cereal Sci.

[30] Kutman U.B., Yildiz B., Cakmak I. (2011). Improved nitrogen status enhances zinc and iron concentrations both in the whole grain and the endosperm fraction of wheat. J. Cereal Sci.

[31] Re R., Pellegrini N., Proteggente A., Pannala A., Yang M., Rice-Evans C. (1999). Antioxidant activity applying an improved ABTS radical cation decolorization assay. Free Radic. Biol. Med.

[32] Oyaizu M. (1986). Studies on products of the browning reaction. Antioxidative activities of browning reaction products prepared from glucosamine. Jpn. J. Nutr.

[33] Wang T., Jónsdóttir R., Ólafsdóttir G. (2009). Total phenolic compounds, radical scavenging and metal chelation of extracts from Icelandic seaweeds. Food Chem.

